# Design, Synthesis, Antimicrobial, and Anticancer Activities of Acridine Thiosemicarbazides Derivatives

**DOI:** 10.3390/molecules24112065

**Published:** 2019-05-30

**Authors:** Rui Chen, Lini Huo, Yogini Jaiswal, Jiayong Huang, Zhenguo Zhong, Jing Zhong, Leonard Williams, Xing Xia, Yan Liang, Zhenshuo Yan

**Affiliations:** 1 College of Pharmacy, Guangxi University of Chinese Medicine, Nanning 530222, China; 58251323@163.com (R.C.); hjyshjy@126.com (J.H.); gxtcmuzzg@163.com (Z.Z.); zhongjing1212@163.com (J.Z.); xiaxing66@163.com (X.X.); yanzhenshuo8@163.com (Z.Y.); 2 Faculty of Chinese Medicine Science, Guangxi University of Chinese Medicine, Nanning 530222, China; 3 Center for Excellence in Post-Harvest Technologies, North Carolina A&T State University, The North Carolina Research Campus, 500 Laureate Way, Kannapolis, NC-28081, USA; yoginijaiswal@gmail.com (Y.J.); llw@ncat.edu (L.W.); 4 College of Pharmacy, Guangxi Medical University, Nanning 530021, China; vincyliang@163.com

**Keywords:** acridine, thiosemicarbazides, anticancer, antimicrobial

## Abstract

**Background:** Acridine and thiourea derivatives are important compounds in medicinal chemistry due to their diverse biological properties including anticancer and antimicrobial effects. However, literature reveals some side effects associated with use of acridines. It is suggested that hybrid molecules may reduce the side effects and enhance the beneficial properties due to synergistic activity. The objectives of the present study are to synthesize and evaluate the anticancer and antimicrobial properties of new hybrids of acridine thiosemicarbazides derivatives. **Results:** The structures of the synthesized compounds **4a–4e** were elucidated by MS and NMR spectra. In antimicrobial assay, Compound **4c** exhibited potent antimicrobial activity compared to the other four compounds. In anticancer studies, we observed that compounds **4a**, **4b**, **4d** and **4e** exhibited high cytotoxicity against the MT-4 cell line, with IC50 values of 18.42 ± 1.18, 15.73 ± 0.90, 10.96 ± 0.62 and 11.63 ± 0.11 μM, respectively. The evaluation of anticancer effects, and the associated mechanism reveals that, the anticancer activities may be related to Topo I inhibitory activity, apoptosis and cell-cycle. Molecular docking studies revealed that the presence of planar naphtho-fused rings and a flexible thiourea group together, could improve DNA-intercalation and inhibition of DNA-Topo I activity. **Conclusions:** The results of this study demonstrate that the rational design of target derivatives as novel antimicrobial or antitumor leads is feasible.

## 1. Introduction

Natural and synthetic acridine derivatives are a series of heterocyclic compounds that are of considerable interest for medicinal chemists and are widely used as anti-inflammatory, antibacterial and antitumor agents [1]. Acridine is a potential compound for developing new anticancer drugs due to its planar structure that can strongly bind to DNA [2]. The cytotoxicity effect of most clinically useful DNA-intercalating agents involves the inhibition of the enzyme DNA-topoisomerase I or II [3]. Acridine compounds are able to inhibit topoisomerase I and II enzymes, render a DNA damage, disrupt DNA repair and replication, and induce cell death [4,5,6]. Amsacrine (m-AMSA, **1**) and *N*-[(2-dimethylamino)ethyl] acridine-4-carboxamide (DACA, **2**) are the most common acridine DNA-topoisomerase inhibitors. For example, m-AMSA and its analogue (**3**) have been clinically used for treatment of leukemia due to their DNA-intercalation activity and inhibition of the enzyme DNA-topoisomerase I or II [7,8,9]. DACA is a new DNA-intercalating agent with inhibitory activity against the enzymes topoisomerase I and topoisomerase II. It is currently in clinical trial as an anticancer drug for patients with non-small cell lung cancer and advanced ovarian cancer [10]. Compounds or derivatives with above-mentioned acridine structure, are antitumor cytotoxic agents with DNA-intercalative properties. Their specific characteristics include; (1) the presence of the planar acridine-platform and, (2) one or two flexible substituent groups. 
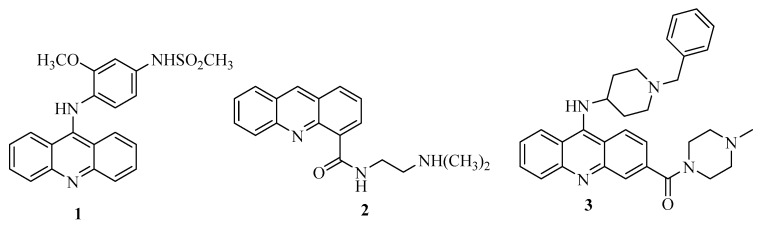


Thiourea and sulfonamide derivatives have gained attention of medicinal chemists due to their wide range of biological activities, which include; antitumor, antiviral, antimicrobial, antiparasitic, and fungicidal effects [11,12,13,14,15,16]. Unfortunately, the use of some acridines has been limited due to issues such as side effects, drug resistance and poor bioavailability [3]. There is a rising need for development of new acridine compounds and derivatives possessing potent antitumor activities, but with reduced side effects. A new approach to reduce drug resistance, is the synthesis of hybrid molecules with enhanced activities [17]. Hybridization of two bioactive molecules often leads to increased activity due to synergistic effects. A strong prevalence of intramolecular hydrogen bonding between the thiourea group and the receptor pocket helps in improving the bioactivities of compounds [18].

**Figure 1:**
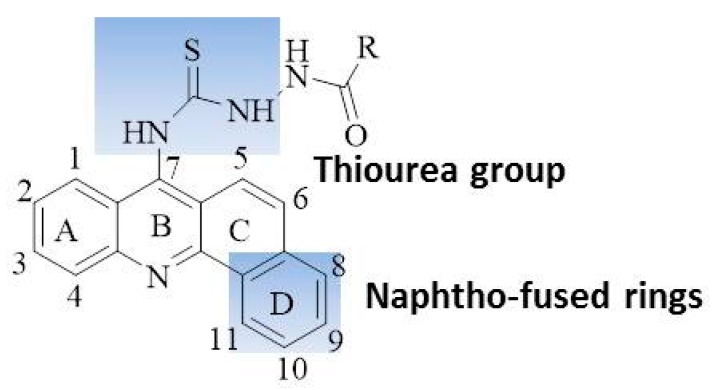
Hybrids of acridine and thiourea group giving acridine thiosemicarbazides.

In light of the above-mentioned considerations for anticancer agents with enhanced activity and reduced side effects, the objective of this study was to synthesize potential anti-cancer compounds that are hybrids of acridine and thiourea group (Figure 1). A new naphtho-fused ring was added in the acridine structure for increasing the DNA imbedding by their planar platform. The objectives of the study also include an investigation of the mechanism of action of these compounds for topo I inhibition, cell apoptosis and selectivity for the cellular cycle. As acridine derivatives are reported to exhibit other pharmacological properties such as antibacterial effects, the synthesized compounds were screened for antibacterial activity.

## 2. Results and Discussion

### 2.1. Chemistry

**Scheme 1:**
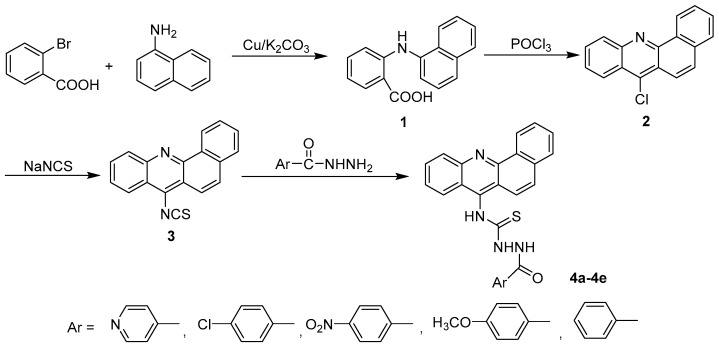
The synthesis of acridinyl aroyl thiourea derivatives (**4a–4e**).

The general synthetic approach for acridinyl acylthiourea derivatives (4) is illustrated in Scheme 1. Naphtho-fused acridinyl skeleton (2) was obtained from the classic synthetic methods of acridine. Isothiocyanate 3 was obtained from Compound 2 with nucleophilic substitution. The target compounds 4a–4e were synthesized by the condensation of aryl hydrazides with naphtho-fused acridinyl isothiocyanates (3), and their yields ranged from 31.0 to 68.2%. The substituents on aromatic ring influenced the yields of the product. It was found that, electron-donating group helped in increasing the nucleophilicity of the amino group of aryl hydrazides. Therefore, 4d (when substituent on aromatic ring was methoxyl group) obtained a yield of up to 68.2%. Solvent selection was critical in the last step. Ethyl alcohol was found to be the best solvent for refluxing. This is because, after cooling to room temperature the pure products 4a-4e formed easily, and required no further purification. When other solvents (such as acetonitrile) were used, both yield and purity were negatively affected.

The structures of compounds were elucidated by MS, ^1^H NMR and ^13^C NMR (All the copies of ^1^H NMR, ^13^C NMR and MS for all compounds are available in Supplementary Materials). All spectral data were in accordance with the assumed structures and are summarized in Table 1. In ^1^H NMR spectra, the three N-H protons of acylthiosemicarbazides **4a**–**4e** were observed at 10.82–11.36, 10.49–10.57 and 10.15–10.31 ppm. Their ^13^C NMR spectra showed the characteristic carbon signals at δ 183.0 and δ 165.0, nearby attributed to C=S and C=O. The ESI-MS indicated that the molecular weights were in accordance to the calculated value.

### 2.2. Biological Study

#### 2.2.1. Antibacterial Activity 

Evaluation of antibacterial activity against a group of pathogenic microorganisms, including Gram-positive bacteria (*Staphylococcus aureus*), Gram-negative bacteria (*Shigella Castellani, Escherichia coli and Pseudomonas aeruginosa*), and fungi (*Candida albicans*), was carried out for the newly synthesized compounds. The results of the study are shown in Table 2. Antimicrobial activities are presented as the minimum inhibitory concentrations (MICs), which is the lowest concentration of the examined compound that resulted in more than 80% growth inhibition of the microorganism. In case of Gram-positive bacteria, **4a** and **4c** showed significant activity against *Staphylococcus aureus* with MIC at 10 μM. Except **4d**, all compounds exhibited promising activity against *Pseudomonas aeruginosa*. In addition, **4c** and **4e** exhibited some antifungal activity with MICs of 10 and 20 μM, respectively. In general, **4c** exhibited more potent antimicrobial activities compared to other compounds. Therefore, we infer that among the compounds studied, **4c** can be taken as the lead compound for the development of novel antimicrobial agent.

#### 2.2.2. Anti-Proliferative Activity Screening

All the synthesized compounds were evaluated for their cytotoxicity effect in five human cancer cell lines. The cell lines include; leukemia cell HL-60, acute lymphoblastic leukemia cell MT-4, cervical cancer cell Hela, hepatocellular carcinoma cell HepG2 and breast cancer cell MCF-The obtained IC_50_ of the synthesized compounds compared to the reference drug are shown in Table 3. The anti-proliferative activity results indicate that some of the target compounds possess notable anti-cancer properties. Interestingly, the highest activity in MT-4 is displayed by compounds **4a**, **4b**, **4d** and **4e** (IC_50_ = 18.42 ± 1.18, 15.73 ± 0.90, 10.96 ± 0.62 and 11.63 ± 0.11 μM), respectively. Compound **4d** exhibited relatively high activity in HepG2 (IC_50_ = 29.05 ± 1.87 μM) cells compared to the Compound **4a** (IC_50_ = 32.96 ± 0.81 μM). All compounds were proved inactive in HL-60 and MCF-7 cell lines. 

#### 2.2.3. Evaluation of Topo I Inhibitory Activity and Molecular Docking Study 

**Figure 2:**
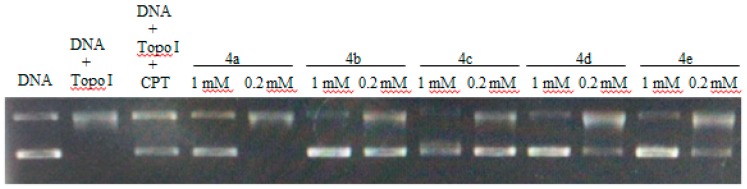
DNA-topo I inhibitory activity of **4a**–**4e** using camptothecin (CPT) (0.2 mM) as a positive control.

DNA-topo I is an important enzyme in all living organisms, and it participates in many cellular metabolic processes, such as replication, transcription, recombination, and repair [19]. DNA topoisomerases have been established as molecular targets of anticancer drugs. The topo I inhibitory activity assay was carried out using the supercoiled DNA unwinding method [20]. A quantitative assay was carried out to assess the relative topo I inhibitory potency of the compounds and compared with the known topo I inhibitor camptothecin (CPT). The topo I inhibitory activity of the compounds is depicted in Figure 2. Similar to CPT, compounds **4b**, **4d** and **4e** showed topo I inhibitory activity at 0.2 mM concentration. Compound **4a** showed significant topo I inhibitory activity only at 1 mM. In many cases, the topo I inhibitory activity did not correlate well with the cytotoxicity. Compound **4d** showed potent cytotoxicity in most of the cell lines but moderate topo 1 inhibitory activity, which may be related to other anticancer mechanisms.

**Figure 3:**
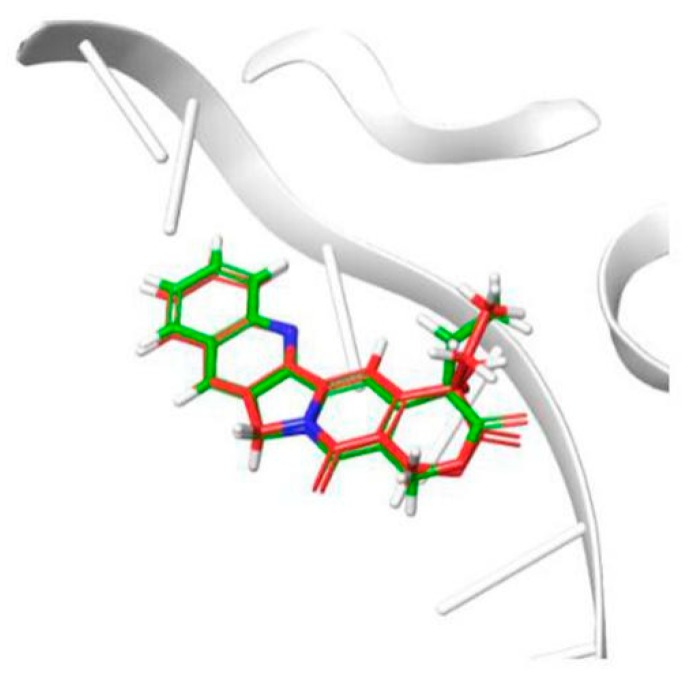
Result of validation for Topo I inhibitor camptothecin (CPT), inside the enzyme active site (derived from PDB ID 1T8I).

To investigate the binding mode of potent inhibitor with human DNA-Topo I complex, molecular docking study was carried out by using Surflex-Dock in Sybyl-X 2.0. The 1T8I (PDB code) structure in Protein Data Bank was improved and used in the study. To validate the molecular docking approach used in this study, the crystallographic pose of CTP, derived from the DNA-Topo I complex structure (PDB ID 1T8I), and the top-ranked docking pose obtained in this study, were compared. The results indicated in Figure 3, depict the superposition of the two binding poses of CTP, inside the DNA-Topo I binding site. This superposition results in a RMSD (root-mean-square deviation) of superposition of 0.54 Å. The obtained RMSD value is far below the well-established tolerance level of 2.0 Å, thus validating the adopted docking methodology [21,22]. 

**Figure 4:**
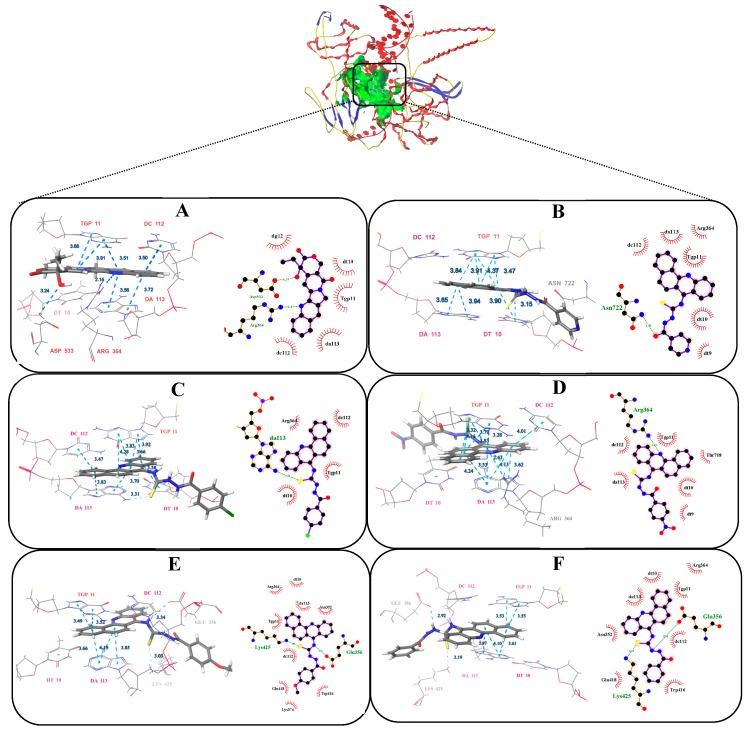
The best pose of the binding mode of compounds with DNA-Topo I complex (PDB:1T8I). (**A**: **CPT**, **B**: **4a**, **C**: **4b**, **D**: **4c**, **E**: **4d F**: **4e**).

After running Surflex-Dock, the binding affinities of protein-ligand complexes were obtained and expressed as total score. The number of H-bonds and binding residues are indicated in Table 4. Docking results indicate that compounds (**4a**–**4e**) occupy the same binding site as that of CPT (Figure 4). The benz[c]acridine ring could take effect with the same base pair TGP11, DA113 and DC112, by π-π stacking force. Besides, the nitrogen and sulphur atoms in thiocarbamide moiety of compounds **4d** and **4e**, were found to have two hydrogen bonding interactions with the adenosine GLU356 and LYSAll these interactions helped them to anchor in the binding site of the protein and have good docking score as CPT, with total scores of 9.62, 10.31 and 10.27, respectively. Only one hydrogen bonding was formed in **4a**–**4c**. As predicted, **4d** and **4e** exhibited remarkable topo I inhibitory activities. 

#### 2.2.4. Apoptosis and Cell-Cycle Analysis

The initiation of apoptosis and stages of cell cycle play an important role in progression of cancer. These two factors are considered crucial in cancer therapy [23,24]. The most active compound, **4d**, was selected to study its effect on apoptosis and cell-cycle profile. As MT-4 cells were most sensitive to the activity of tested compounds, they were selected for testing Compound **4d** for its effect on apoptosis and cell-cycle profile.

**Figure 5:**
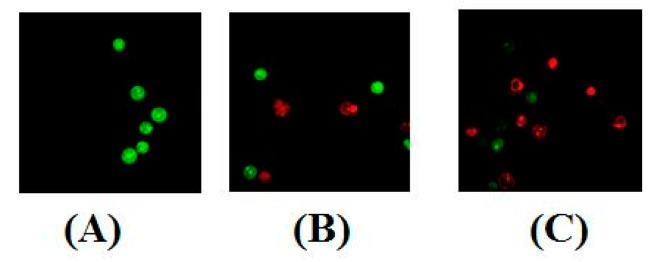
AO/EB staining of Compound 4d in MT-4 cells. (**A**) Cells not treated with **4d** (control) and (**B**) cells treated with Compound **4d** at 12.50 μM and, (**C**) cells treated with Compound **4d** at 25 μM concentration.

Determination of apoptosis was carried out using two methods viz. nuclear morphology determination (by use of dyes) and flow cytometry analysis (by determining apoptosis ratios). Nuclear morphological changes indicating tumor cell apoptosis were detected by using acridine orange (AO)/ethidium bromide (EB) stains. The use of these dyes facilitated a clear distinction between normal cells, early and late apoptotic cells, and necrotic cells [25]. No significant apoptosis was detected in the negative control group (Figure 5A). As evident in Figure 5B,C, the nuclei of MT-4 cells were markedly stained as yellow green or orange and the morphology displayed pycnosis, membrane blebbing and cell budding when treated with Compound **4d** for 24 h. These findings indicate that Compound **4d** could induce MT-4 cells apoptosis. With increasing concentrations, the number of apoptotic cells increased.

**Figure 6:**
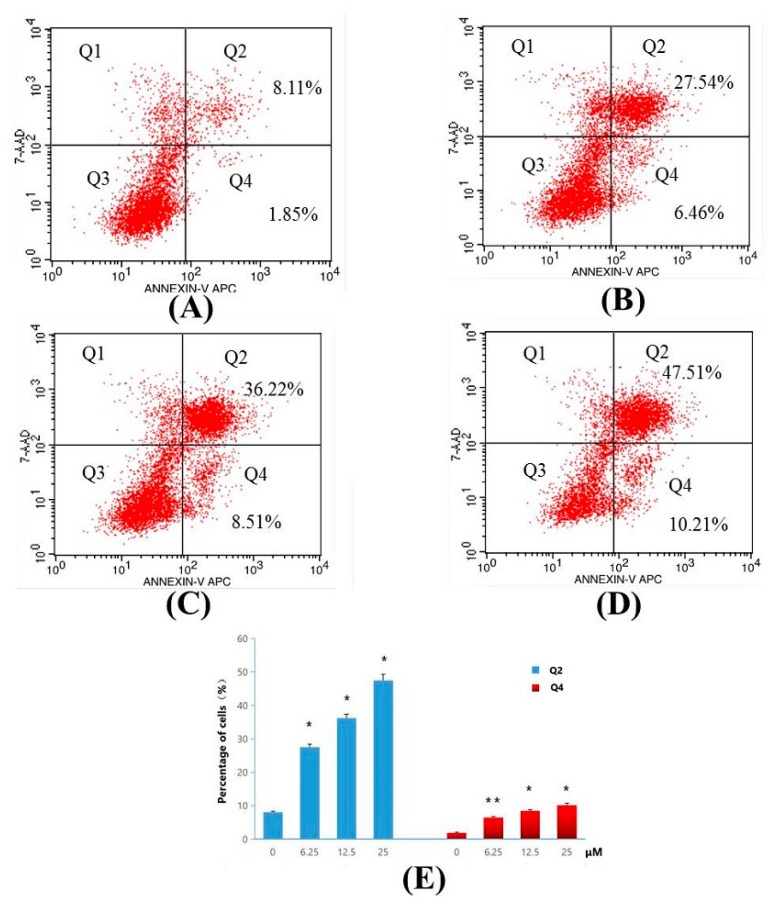
Apoptosis ratio detection of Compound **4d** by Annexin V/PI assay. (**A**) Not treated with Compound **4d** (control) for 48 h and, (**B**) treatment with Compound **4d** at 6.25 μM, (**C**) treatment with Compound **4d** at 12.50 μM and (**D**) treatment with Compound **4d** at 25 μM for 48 h each, respectively, (**E**) The percentage of quadrants Q2 and Q4 was shown as mean ± SD. Data is the representative of three independent experiments. * *p* < 0.05, ** *p* < 0.01.

For determination of apoptosis ratios of Compound **4d** in early and late stage apoptosis, flow cytometry analysis was used. MT-4 cells were treated with 6.25, 12.5 and 25 μM concentrations of **4d**, and the apoptosis ratios were obtained. The four quadrant images describing, damaged, necrotic/apoptotic, normal and early apoptotic cells were observed in quadrants Q1, Q2, Q3, and Q4, respectively during flow cytometry analysis [26]. The sum of quadrants Q2 and Q4 is used to calculate the apoptosis ratio. As seen in Figure 6, the apoptosis ratios of Compound 4d measured at different concentration points were found to be 34.00% (6.25 μM), 44.73% (12.5 μM) and 57.72% (25 μM), respectively. The apoptosis ratio for control was found to be 9.96%. The obtained results thus indicate that Compound **4d** suppressed cell proliferation by inducing apoptosis, and the apoptosis of MT-4 cells treated with Compound **4d** increased gradually in a concentration-dependent manner.

**Figure 7:**
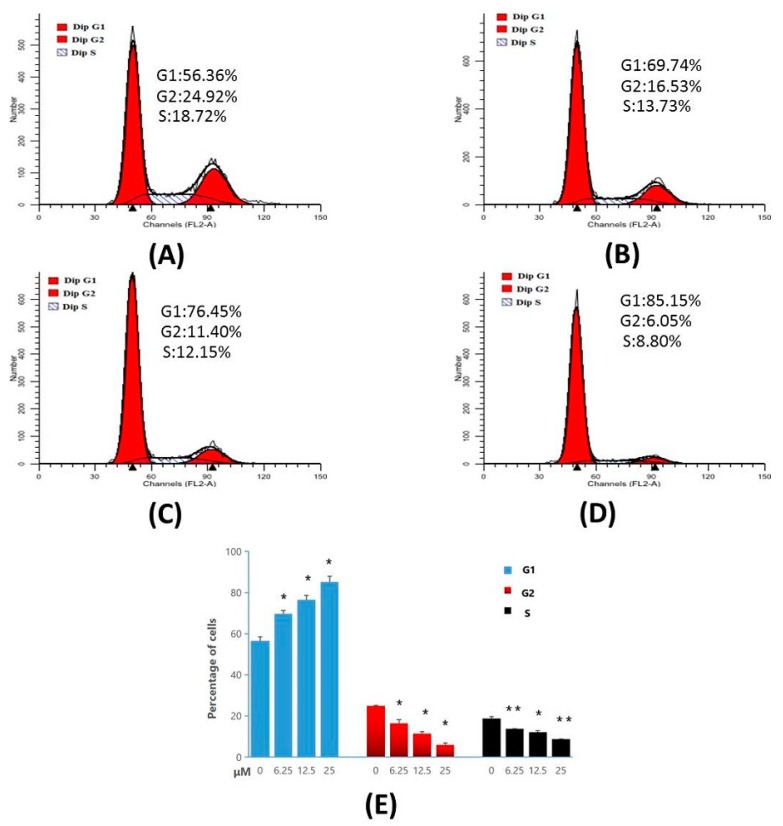
Cell-cycle analysis of MT-4 cells treated with Compound **4d**, by flow cytometry. (**A**) Not treated with Compound **4d** (control) for 48 h (**B**) treatment with Compound **4d** at 6.25 μM, (**C**) treatment with Compound **4d** at 12.50 μM and, (**D**) treatment with Compound **4d** at 25 μM for 48 h each, respectively, (**E**) The percentage of each population was shown as mean ± SD. Data is the representative of three independent experiments. * *p* < 0.05, ** *p* < 0.01.

The results of cell-cycle analysis performed in MT-4 cells, are represented in Figure 7. The results indicate that, Compound **4d** exhibited 69.74%, 76.45%, and 85.15% of cell accumulation in G1 phase at 6.25 µM, 12.50 µM and 25.00 µM concentrations, respectively. However, in control (untreated cells) only 56.36% of cell accumulation in G1 phase was observed. These results indicate that Compound **4d** induced a significant cell-cycle arrest in G1 phase in a concentration-dependent manner, compared to the control cells.

## 3. Materials and Methods 

All chemicals were reagent grade and were purchased from commercial sources. All yields refer to isolated products after purification. NMR spectra were measured on a Bruker DRX-500 (^1^H: 400 MHz, ^13^C: 100 MHz) (Rheinstetten, Germany) using CDCl_3_ and DMSO-*d*_6_ as solvents. Chemical shifts (d) are expressed in parts per million (ppm), and *J* values are given in hertz (Hz). The mass spectra were obtained on a Thermo Fisher LCQ Fleet (ESI) instrument (Waltham, MA, USA). Melting points were determined using an Micro Melting-point Apparatus X-4A (Precision Instrument Co., Ltd., Shanghai, China) and were uncorrected.

### 3.1. Synthesis Methods

#### 3.1.1. Synthesis of *N*-naphthyl-o-aminobenzoic Acid (**1**)

A mixture of 10.5 g bromobenzoic acid (26 mmoL), 9.8 g naphthylamine (68 mmoL), 15 g potassium carbonate (36.2 mmoL) and 0.6 g copper powder (9.4 mmoL) in isopentanol (60 mL) was stirred under reflux at 140 °C for 2 h. After the reaction, the isopentanol was removed under vacuum and the residue was diluted with 1200 mL water and then was stirred at 80 °C for 20 min. It was immediately filtered while hot. The water phase was acidified by hydrochloric acid upto pH 2, to facilitate precipitation. The precipitate was then filtered and recrystallized using chloroform to obtain greenish yellow solid, yield 79.0%, m.p. 204.8–208.4 °C.

#### 3.1.2. Synthesis of 7-chlorine benz[c]acridine (**2**)

In a 250 mL round-bottom flask, Compound **1** 3.36 g (13.8 mmoL) and phosphoryl chloride (9.18 mL) were added. The reaction mixture was stirred and heated to about 85–90 °C for 15 min. If liquid flooding occurred, the mixture was immediately removed from the hot bath until flooding ceased. The mixture was stirred for 2.5 h at 135–140 °C. On completion of the reaction, the solvent was removed from under pressure and the residue was poured into a mixture of 13.5 mL aqua ammoniac, 34.0 g crushed ice and 15 mL chloroform. Then, the water phase was extracted three times with 20 mL chloroform. The chloroform phase was dried with anhydrous calcium chloride overnight, filtered, evaporated and recrystallized with acetone to obtain faint yellow needle crystals, yield 67.5%, m.p. 140.1–141.7 °C.

#### 3.1.3. Synthesis of 7-benz[c]acridine Isothiocyanate (**3**)

7-chlorine benz[c]acridine (1.84 g, 7 mmol) was dissolved in acetone (60 mL), followed by addition of NaSCN (1.14 g, 14 mmol) and tetrabutylammonium bromide (0.21 g, 0.7 mmol). After 1.5 h refluxing with stirring, the reaction mixture was cooled to room temperature. The formed bright yellow needle crystals were fifiltered, washed with water, and dried under vacuum. Yield 82.0%, m.p. 230–233 °C. ESI-MS: *m*/*z*: 287 [(M + H)^+^]; ^1^H-NMR (CDCl_3_, 600 MHz): δ 9.47 (d, *J* = 7.8 Hz, 1H), 8.40 (d, *J* = 8.6 Hz, 1H), 8.32–8.22 (m, 1H), 8.01 (d, *J* = 9.2 Hz, 1H), 7.89 (dd, *J* = 8.4, 5.9 Hz, 2H), 7.83–7.75 (m, 3H), 7.71 (ddd, *J* = 8.1, 6.7, 1.2 Hz, 1H); ^13^C-NMR (CDCl_3_, 150 MHz): δ 147.96, 147.61, 140.26, 133.61, 131.12, 130.32, 129.61, 129.32, 128.13, 127.89, 127.15, 125.45, 122.68, 120.39, 120.11.

#### 3.1.4. General Procedure for the Synthesis of 1-Aryl-4-(7-benz[c]acridinyl) Thiosemicarbazides Derivatives **4a**–**e**

To a solution of 7-benz[c]acridine isothiocyanate **3** (0.2 g, 0.7 mmol) in absolute ethyl alcohol (50 mL), the appropriate substituted hydrazides (0.8 mmol) were added and the reaction mixture was refluxed until the reactants were consumed. The formed precipitate was filtered off, washed with a small amount of hot ethyl alcohol and dried at room temperature to give pure products **4a**–**e**.

1-Pyridyl-4-(7-benz[c]acridinyl) Thiosemicarbazides (**4a**): Orange-yellow powder, yield 46.7%, m.p. 177.4–180.9 °C.
1-(4-chlorin-phenyl)-4-(7-benz[c]acridinyl) Thiosemicarbazides (**4b**): Orange-yellow powder, yield 34.0%, m.p. 203.3–203.9 °C.
1-(4-nitro-phenyl)-4-(7-benz[c]acridinyl) Thiosemicarbazides (**4c**): Orange-red powder, yield 31.0%, m.p. 238.7–240.1 °C.
1-(4-methoxy-phenyl)-4-(7-benz[c]acridinyl) Thiosemicarbazides (**4d**): Orange-yellow powder, yield 68.2%, m.p. 184.2–187.4 °C.
1-phenyl-4-(7-benz[c]acridinyl) Thiosemicarbazides (**4e**): Orange-yellow powder, yield 58.6%, m.p. 181.4–182.9 °C

### 3.2. Biological Activity

#### 3.2.1. Antimicrobial Activity

The in vitro anti-bacterial activity testing of the thiosemicarbazides derivatives (**4a**–**4e**) was carried out by tube dilution method [27] using Gram-positive (*Staphylococcus aureus*) and Gram-negative bacteria (*Shigella Castellani,* and *Escherichia coli*). The antifungal effect was screened against *Candida albicans*. Dimethyl sulfoxide was used as a solvent to prepare the stock solutions of the test and reference compound (streptomycin). For bacteria and fungi, double strength nutrient broth I.P. and sabouraud dextrose broth I.P., respectively were used for preparing serial dilutions of the test and reference compound. [28]. The incubation period used for inoculated plates of bacteria and fungi were 24 h and 48 h, respectively at 37 °C. The results were recorded in terms of the minimum inhibitory concentration (MIC). The MIC was defined as the lowest concentration of the tested compound inhibiting the visible growth of each microorganism. Each test was carried out in triplicate. 

#### 3.2.2. Antiproliferative Activity

##### MTS Assay

Cytotoxicity in cells was investigated by MTS method [29]. A panel of five human cancer cell lines including leukemia cell HL-60, acute lymphoblastic leukemia cell MT-4, cervical cancer cell Hela, hepatocellular carcinoma cell HepG2 and breast cancer cell MCF-7, were used. All these tumor cell lines were obtained from Kunming Institute of Botany. Briefly, cells were seeded on 96-well plates at a density of 4 × 10^3^–15 × 10^3^ cells/well, incubated for 24 h at 37 °C. These were then treated with test drugs and cisplatin for 48 h. To determine the live cell numbers, MTS (3-(4,5-dimethylthiazole-2-yl)-5-(3-carboxymethoxyphenyl)-2-(4-sulfophenyl)-2*H*-tetrazolium) (Promega, Madison, WI, USA) was added to the cells and allowed to develop for 2–4 h. Colorimetric measurements were taken at 492 nm. The drug concentrations resulting in 50% inhibition of cell growth (IC_50_) were determined by Reed and Muench method.

### 3.3. Topo I Inhibitory Activity 

Supercoiled pBR32 DNA was used a substrate to determine the Topo I catalytic activity [29]. Commercial samples of Topo I and pBR322 were obtained from Takara Biotechnology (Dalian) Co.,Ltd.Takara Bio Inc. (Dalian, China). The buffer solutions were prepared using 500 mM KAc, 200 mM Tris-Ac, 100 mM Mg(Ac)_2_ and 1 mg/mL BSA. The procedure used for determination of enzyme inhibitory activity was similar to one reported by Xu et al. [30].

### 3.4. Molecular Docking

The Surflex-Dock in Sybyl-X version 2.0 by Tripos Associates (L.P. St. Louis, MO, USA) was used for molecular docking. This system performs molecular docking functions aided by the generation of an idealized active site (Protocol), consisting of dummy atoms that guide the docking process. The crystal structures of DNA-Topo I complex was downloaded from the Research Collaboration for Structural Bioinformatics (RCSB) website (www.rcsb.com) (PDB ID: 1T8I). The proteins were then imported into the Surflex-Dock, and prepared for docking using the biopolymer preparation tool according to the following criteria: H-Addition, H-Bond, removal of water molecules, termini treatment, charge, protonation, type of histidines. 

The structures of the compounds were sketched using software of ChemBioOffice version 14.0 by PerkinElmer (Waltham, MA, USA). The alignments of the training set molecules were derived by using FlexS in Sybyl–X. All values were filled with valence, and Gasteiger–Marsili charges were calculated for each compound. Ultimately, ligand docking under the Surflex–Dock GeomX precision was performed to generate grid of protein and ligands. The docking results were then imported into the LigPlot^+^ 2.1, and the combination of compounds and proteins were discussed by H-Bond, conjugate action, and hydrophilic or hydrophobic action.

### 3.5. General Procedure for AO/EB Staining 

Apoptosis was determined by nuclear morphology. Cells were fixed and stained with AO/EB according to the manufacturer’s instruction (KGA501, KeyGEN BioTECH, Nanjing, China). MT-4 cells were seeded in 6-well plates with a sterile cover slip. The concentration of cells used per well was 5 × 10^5^–6 × 10^6^ cells/2 mL. To facilitate growth, the medium was replaced with fresh medium (RPMI1640) plus 10% fetal bovine serum. The fresh medium was supplemented with Compound **4d** (12.50 μM and 25 μM). Post-treatment, 25 μL of cell suspension was collected on a glass slide by inverting the cover slip and stained with 10 μL of AO/EB stain (100 mg/mL). Finally, stained nuclei were observed immediately under a fluorescence microscope (BX41, Nikon, Tokyo, Japan).

### 3.6. General Procedure for Apoptosis Ratio Determination

For determination of apoptosis ratios by application of flow cytometry analysis, the manufacturer’s protocol (KGA1024, KeyGEN BioTECH, Nanjing, China) for Annexin-V APC/7-AAD double-stain assay was used. Briefly, the prepared MT-4 cells (5 × 10^5^ cells/mL) were collected and resuspended in 500 μL of binding buffer containing 5 μL of Annexin V-APC. The suspension was shaken well and 5μL of 7-AAD was added to it. The suspension was then incubated for 5–15 min in the dark at room temperature. Post-incubation, the suspension was immediately analyzed using a flow cytometer (FACS Cali-bur, Becton Dickinson, Mountain View, CA, USA).

### 3.7. General Procedure Cell Cycle 

MT-4 cell cultures were treated with the indicated concentrations of Compound **4d** for 48 h incubation. The cells were washed twice with ice-cold phosphate buffer saline (PBS), fixed and permeabilized with ice-cold 70% ethanol at 4 °C for 2 h. The cells were treated with 100 μL RNase A at 37 °C for 30 min after washing with ice-cold PBS. Then they were stained with 400 μL 1 PI (1 mg/mL), in the dark at 4 °C for 30 min. Cell-cycle analysis was performed by flow cytometry (FACS Verse, BD, USA), at an excitation wavelength of 488 nm.

## 4. Conclusions

The study provides a systematic synthesis scheme for acridine thiosemicarbazides derivatives with possible anti-cancer and anti-microbial activity. Through application of spectral evaluation and in vitro studies, the potential anti-tumor activity of Compound **4d** and anti-microbial activity of Compound **4c** is demonstrated. In the study of pharmacological mechanism, most of the compounds except **4b** exhibited potent topo I inhibitory activity at 0.2 mM. The apoptosis-inducing activity of the representative Compound **4d** in MT-4 cells was studied, and the results revealed that this compound showed clear cell apoptosis-inducing effects. Cell-cycle analysis indicated that Compound **4d** could arrest MT-4 cells in G1 stage. The study demonstrates that the rational design of naphtho-fused acridine thiosemicarbazide derivatives as novel antitumor or anti-microbial leads compared to existing anti-cancer agents is feasible. 

## Figures and Tables

**Table 1:** The spectral data of the compounds (**4a–4e**).

Comp.	Ar	Yield (%)	^1^H NMR (DMSO-*d*_6_) δ ppm (*J* in Hz)	^13^C NMR (DMSO-*d*_6_)	ESI-MS: *m*/*z*: [(M − H)]^−^	Melting Point (°C)
4a	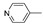	46.7%	11.36 (s, 1H, -NH), 10.55 (s, 1H, -NH), 10.31 (s, 1H, -NH), 9.38~9.41 (m, 1H, ArH), 8.81 (d, 2H, *J* = 4.4Hz, ArH), 8.33 (d, 1H, *J* = 8.8Hz, ArH), 8.21 (d, 1H, *J* = 8.4Hz, ArH), 8.04 (t, 1H, ArH), 7.90~7.97 (m, 5H, ArH), 7.82~7.84 (m, 2H, ArH), 7.73 (t, 1H, ArH)	183.36, 165.46, 150.71, 147.98, 147.88, 142.04, 139.99, 133.88, 131.24, 130.68, 129.99, 129.83, 128.65, 128.09, 128.01, 126.80, 125.34, 125.11, 124.94, 123.22, 122.51, 122.32.	422	177.4–180.9 °C
4b	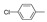	34.0%	11.09 (s, 1H, -NH), 10.52 (s, 1H, -NH), 10.23 (s, 1H, -NH), 9.40 (t, 1H, ArH), 8.33 (d, 1H, *J* = 8.4Hz, ArH), 8.22 (d, 1H, *J* = 8.8Hz, ArH), 8.03~8.09 (m, 3H, ArH), 7.90~7.97 (m,3H, ArH), 7.82~7.84 (m, 2H, ArH), 7.73 (t, 1H, ArH), 7.64 (d, 2H, *J* = 8.4, ArH)	183.49, 165.96, 147.98, 147.87, 142.22, 137.27, 133.89, 131.78, 131.26, 130.62, 130.47,129.95, 129.79, 128.85, 128.62, 127.96, 126.71, 125.39, 125.11, 125.02, 123.25, 122.61.	455	203.3–203.9 °C
4c	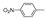	31.0%	11.32 (s, 1H, -NH), 10.57 (s, 1H, -NH), 10.32 (s, 1H, -NH), 9.40 (t, 1H, ArH), 8.40 (d, 2H, *J* = 8.0Hz, ArH), 8.34 (d, 1H, *J* = 8.8Hz, ArH), 8.28 (d, 2H, *J* = 8.4Hz, ArH), 8.21 (d, 1H, *J* = 8.4Hz, ArH), 8.05 (t, 1H, ArH), 7.91~7.97 (m, 3H, ArH), 7.82~7.84 (m, 2H, ArH), 7.74 (t, 1H, ArH)	183.29, 165.42, 149.88, 147.99, 147.88, 142.09, 138.76, 133.89, 131.25, 130.68, 130.05, 129.98, 129.84, 128.65, 128.09, 128.00, 126.80, 125.37, 125.12, 124.95, 123.93, 123.24, 122.52,	466	238.7–240.1 °C
4d	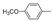	68.2%	10.82 (s, 1H, -NH), 10.49 (s, 1H, -NH), 10.15 (s, 1H, -NH), 9.40 (t, 1H, ArH), 8.33 (d, 1H, *J* = 8.8Hz, ArH), 8.23 (d, 1H, *J* = 8.4Hz, ArH), 7.90~8.05 (m, 6H, ArH), 7.90~7.93 (m, 2H, ArH), 7.82~7.84 (m, 1H, ArH), 7.73 (t, 2H, ArH), 3.83 (s, 3H, OCH_3_).	183.60, 166.39, 147.99, 142.22, 147.86, 142.41, 133.90, 131.27, 130.58, 130.47, 129.92, 128.77, 128.61, 127.93, 126.64, 126.63, 125.45, 125.11, 123.28, 122.72, 113.97, 55.92.	451	184.2–187.4 °C
4e	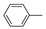	58.6%	11.09 (s, 1H, -NH), 10.52 (s, 1H, -NH), 10.19 (s, 1H, -NH), 9.40 (dd, *J* = 6.1, 3.5 Hz, 1H, ArH), 8.32 (d, *J* = 8.6 Hz, 1H, ArH), 8.25 (d, *J* = 8.5 Hz, 1H, ArH), 8.10 (d, *J* = 7.7 Hz, 2H, ArH), 8.04 (dd, *J* = 6.0, 3.2 Hz, 1H, ArH), 7.99 (d, *J* = 9.3 Hz, 1H, ArH), 7.95~7.88 (m, 2H, ArH), 7.83 (dt, *J* = 6.1, 3.6 Hz, 2H, ArH), 7.72 (t, *J* = 7.6 Hz, 1H, ArH), 7.60 (d, *J* = 7.2 Hz, 1H, ArH), 7.54 (t, *J* = 7.5 Hz, 2H, ArH).	183.51, 166.82, 147.98, 147.86, 142.39, 133.90, 132.93, 131.26, 130.59, 129.92, 129.75, 128.71, 128.61, 127.93, 126.64, 125.43, 125.09, 123.26, 123.26, 122.74	421	181.4–182.9 °C

**Table 2:** Antibacterial activity of 4a–4e presented as the minimum inhibitory concentration (MIC) (μM).

Compounds	Gram-Positive Bacteria	Gram-Negative Bacteria			Antifungal Activity
	*Staphylococcus aureus*	*Shigella Castellani*	*Escherichia coli*	*Pseudomonas aeruginosa*	*Candida albicans*
**4a**	10	>100	>100	10	>100
**4b**	40	20	>100	20	80
**4c**	10	10	20	10	10
**4d**	>100	>100	>100	>100	40
**4e**	40	80	>100	20	20
Streptomycin	2	2	2	2	2

**Table 3:** The IC_50_ value of the synthesized compounds obtained for anti-proliferative activity on different tumor cell lines (μM).

Compounds	HL-60	MT-4	Hela	HepG2	MCF-7
**4a**	>100	18.42 ± 1.18	30.93 ± 1.78	32.96 ± 0.81	>100
**4b**	>100	15.73 ± 0.90	45.68 ± 2.14	54.80 ± 2.95	>100
**4c**	>100	57.59 ± 2.37	59.39 ± 2.56	51.60 ± 2.46	>100
**4d**	59.56 ± 2.54	10.96 ± 0.62	42.04 ± 2.07	29.05 ± 1.87	>100
**4e**	70.19 ± 3.72	11.63 ± 0.11	68.18 ± 3.92	58.99 ± 3.11	>100
cisplatin	3.66 ± 0.14	5.99 ± 0.12	6.65 ± 0.12	14.45 ± 0.49	25.92 ± 1.68

**Table 4:** Interactions between DNA-Topo I (from PDB ID 1T8I) and the compounds.

Compound	Amino Acid	Type	Hydrophobic Residue	Binding Affinity (Total Score)
**4a**	TGP11	π-π	DA113, ARG364, TGP11, DT10, DT9, DC112	8.82
	DA113	π-π		
	DC112	π-π		
	ASN722	Hydrogen Bond		
**4b**	TGP11	π-π	ARG364, DC112, TGP11, DT10	7.82
	DA113	π-π		
	DT10	π-π		
	DC112	π-π		
	DA113	Hydrogen Bond		
**4c**	TGP11	π-π	TGP11, THR718, DT10,DT9, DA113, DC112	8.32
	DA113	π-π		
	DC112	π-π		
	ARG364	Hydrogen Bond		
**4d**	TGP11	π-π	ARG364, DT10, DA113, ASN352, TRP416, LYS374, GLU418, DC112, TGP11	10.31
	DA113	π-π		
	DC112	π-π		
	LYS452	Hydrogen Bond		
	GLU356	Hydrogen Bond		
**4e**	TGP11	π-π	DT10, TGP11, ARG364, DC112, TRP416, GLU418, ASN352,DA113	9.62
	DA113	π-π		
	DC112	π-π		
	LYS425	Hydrogen Bond		
	GLU356	Hydrogen Bond		
CPT	TGP11	π-π	DC112, DA113, TGP11, DT10, DG12	10.27
	DA113	π-π		
	DC112	π-π		
	ASP533	Hydrogen Bond		
	ARP364	Hydrogen Bond		

## Supplementary Materials

All the copies of ^1^H NMR, ^13^C NMR and MS for the title compounds are available online.
