# Supplementary material for: Design, Synthesis, Antimicrobial, and Anticancer Activities of Acridine Thiosemicarbazides Derivatives
Source: Molecules. 2019 May 30;24(11):2065. doi: 10.3390/molecules24112065 (PMC6600397; doi:10.3390/molecules24112065)
Supplement: Supplementary file 1 [file molecules-24-02065-s001.pdf]

Supplementary Materials: Design, Synthesis, Antimicrobial, and Anticancer Activities of Acridine Thiosemicarbazides Derivatives

Rui Chen, Lini Huo, Yogini Jaiswal, Jiayong Huang, Zhenguo Zhong, Jing Zhong, Leonard Williams, Xing Xia, Yan Liang and Zhenshuo Yan

1. Results and Discussion

1.1. Chemistry

Main text paragraph. Mentioning the following Figures.

(1) The MS, ^{1}HNMR, ^{13}CNMR spectrum of **4a**

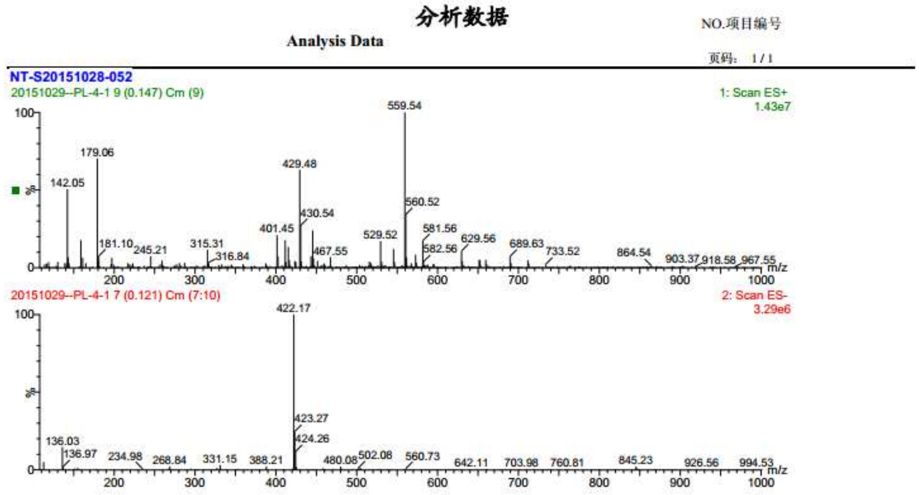

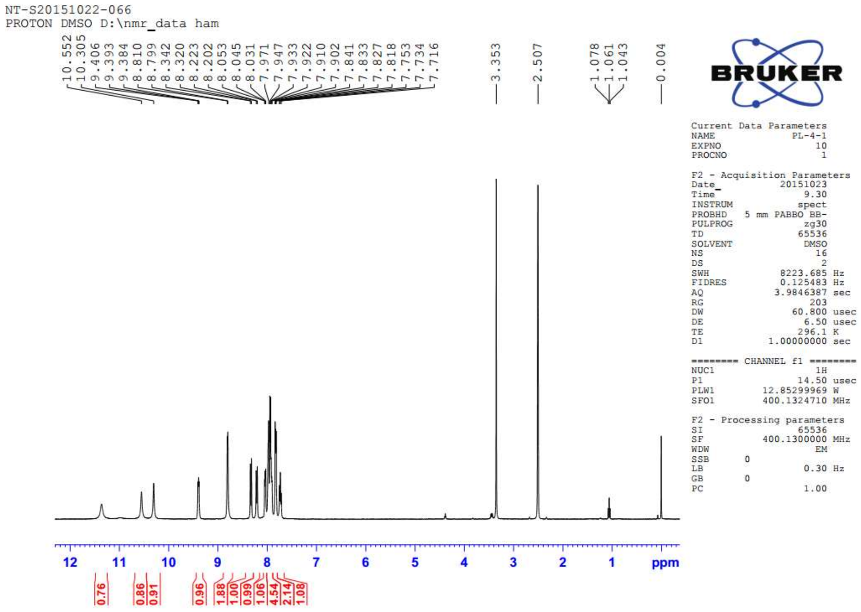

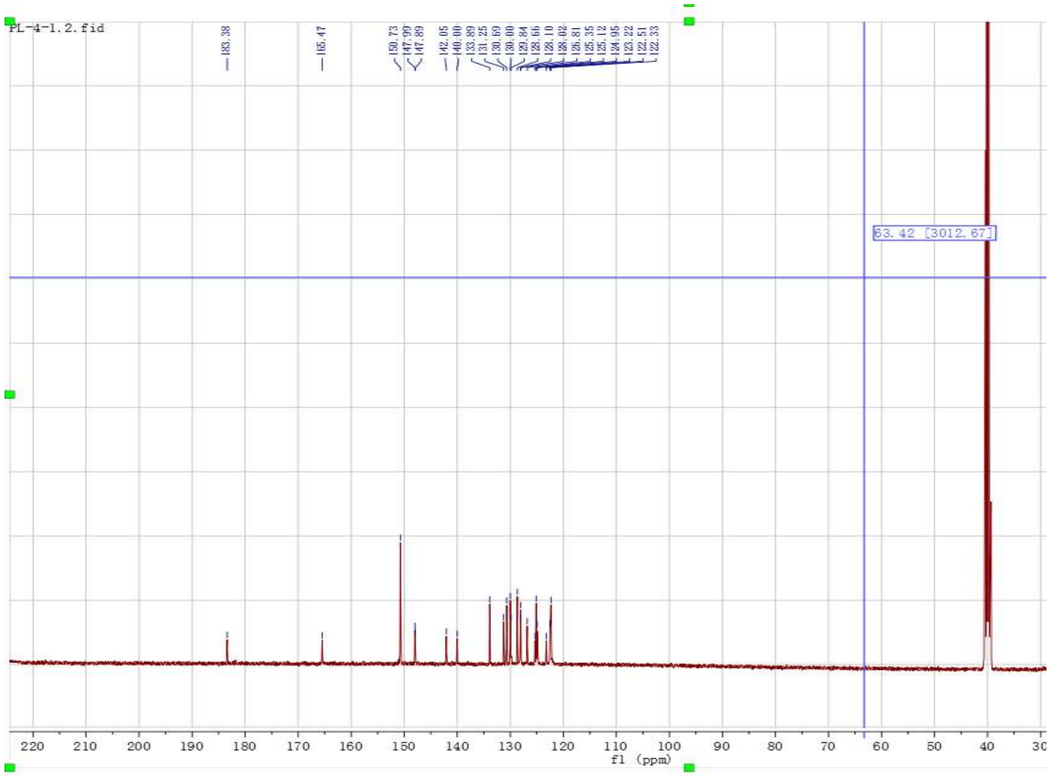

(2) The MS, 1HNMR, 13CNMR spectrum of **4b**

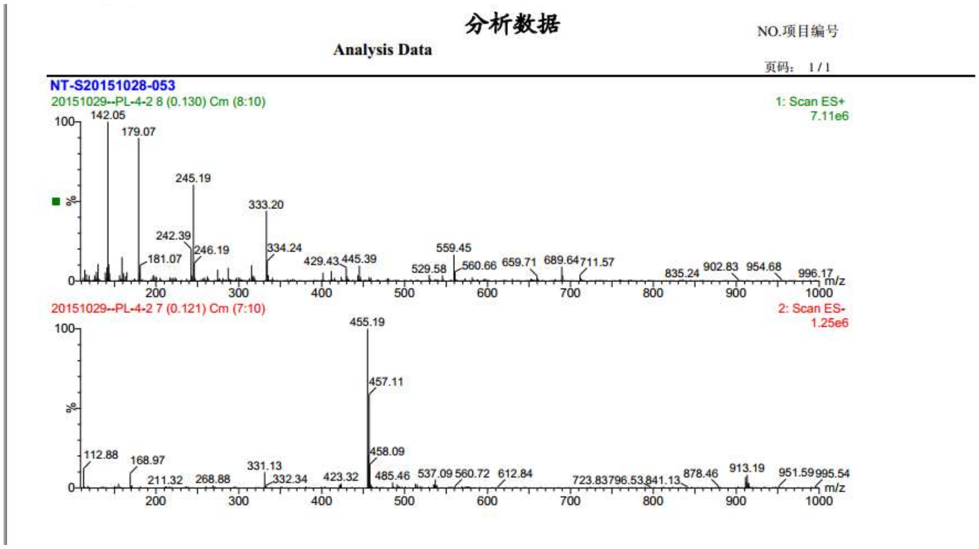

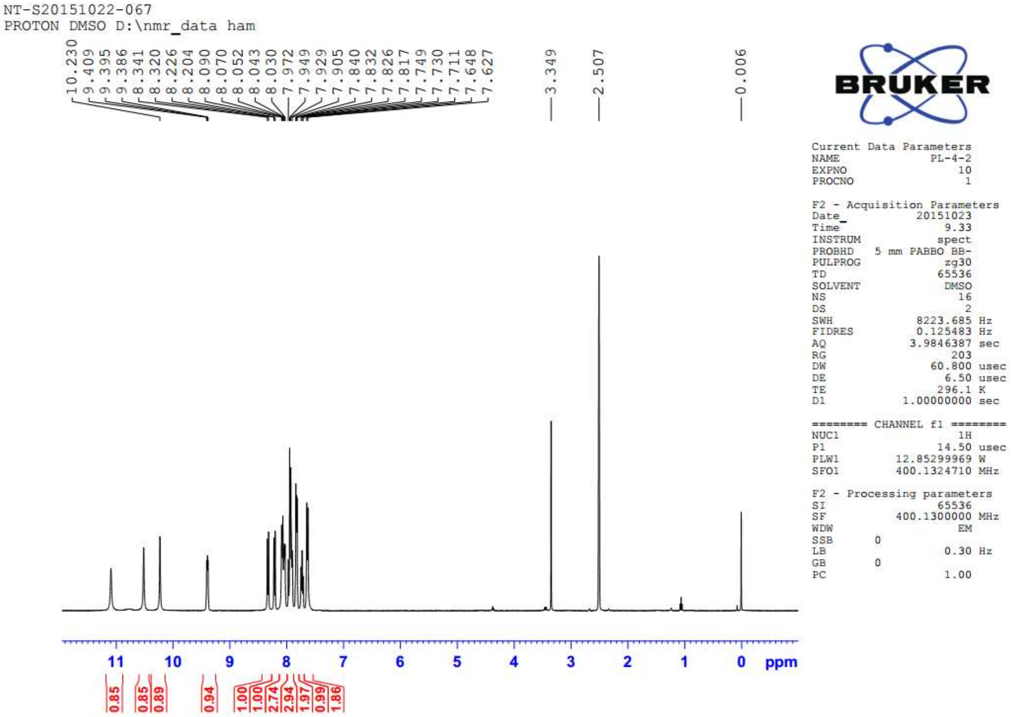

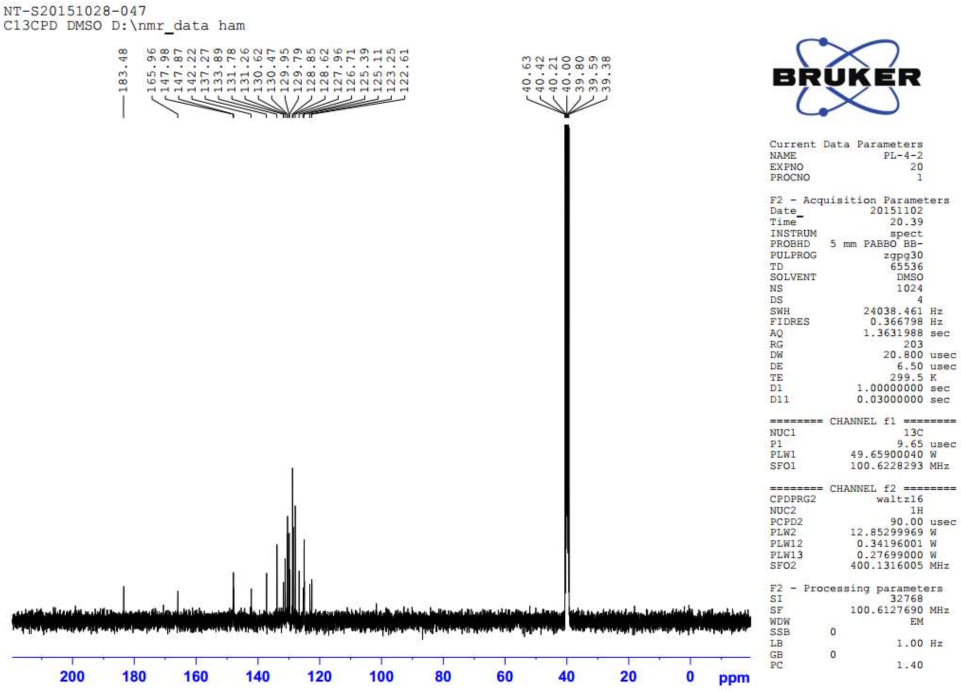

(3) The MS, 1HNMR, 13CNMR spectrum of **4c**

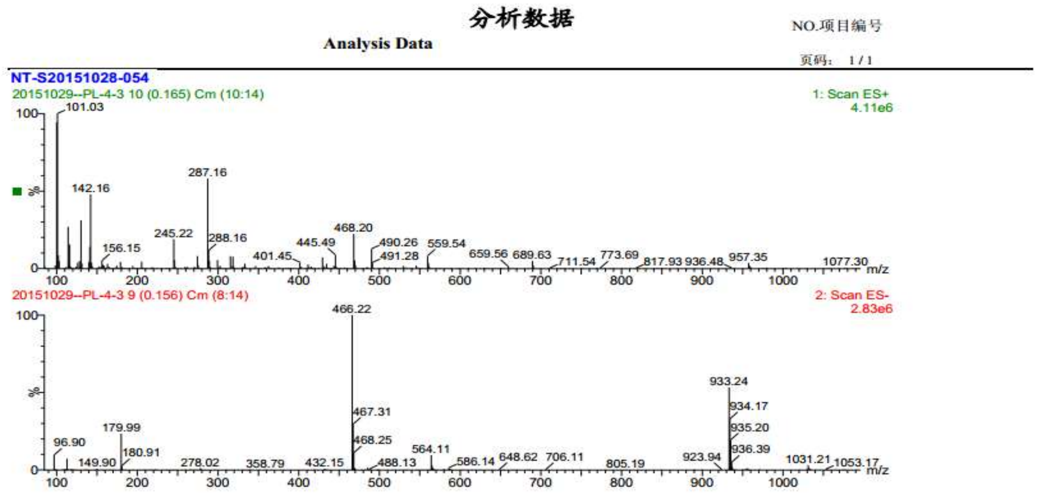
分析数据
Analysis Data
NO.项目编号
页码: 1/1
NT-S20151028-054
20151029--PL-4-3 10 (0.165) Cm (10:14)
1: Scan ES+
4.11e6
101.03
142.16
287.16
245.22
288.16
468.20
445.49
490.26
491.28
559.54
m/z
20151029--PL-4-3 9 (0.156) Cm (8:14)
2: Scan ES-
2.83e6
466.22
933.24
934.17
935.20
936.39
467.31
468.25
179.99
180.91
564.11
m/z

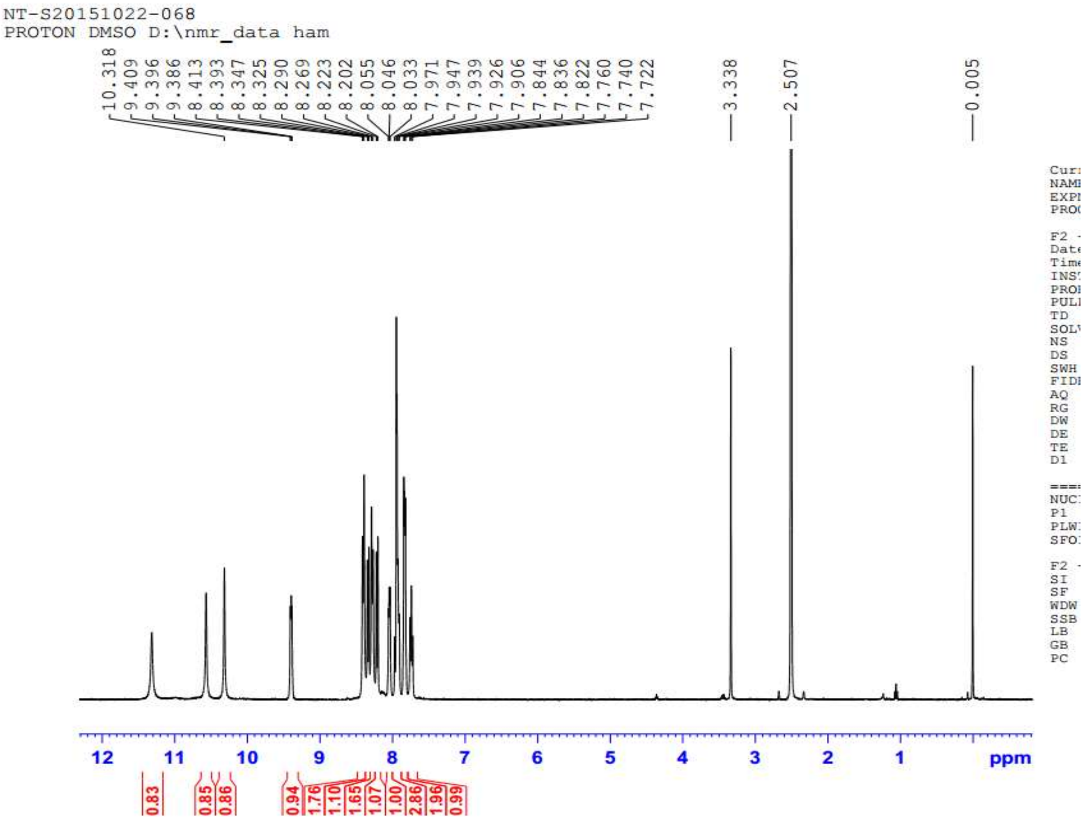
NT-S20151022-068
PROTON DMSO D:\nmr_data ham
10.318
9.409
9.396
9.386
8.413
8.393
8.347
8.325
8.290
8.269
8.223
8.202
8.055
8.046
8.033
7.971
7.947
7.939
7.926
7.906
7.844
7.836
7.822
7.760
7.740
7.722
3.338
2.507
-0.005
ppm
0.83
0.85
0.86
0.94
1.76
1.10
1.65
1.07
1.00
2.86
1.96
0.99

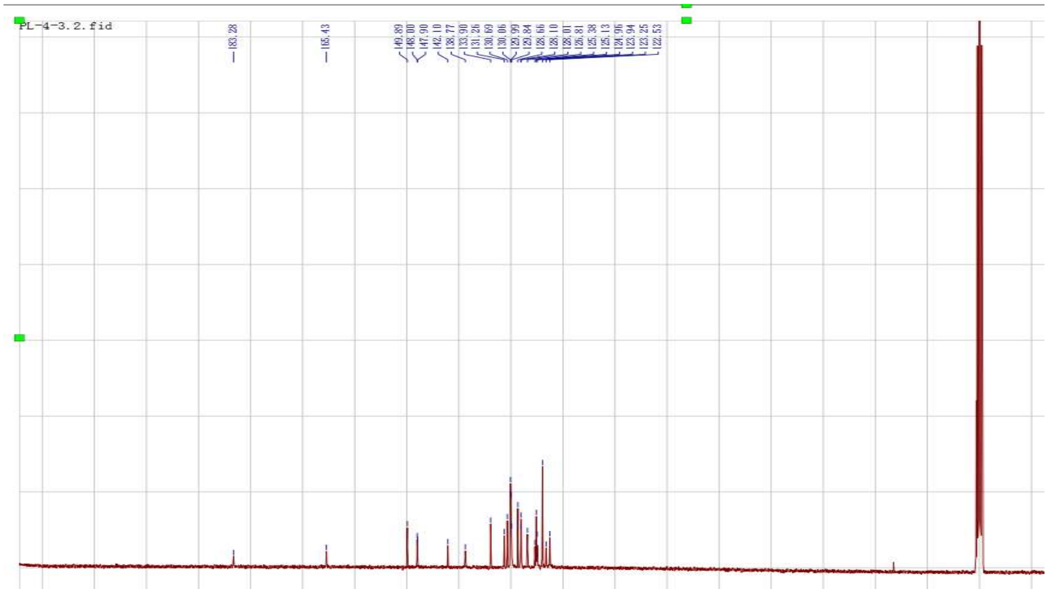

(4) The MS, 1HNMR, 13CNMR spectrum of **4d**

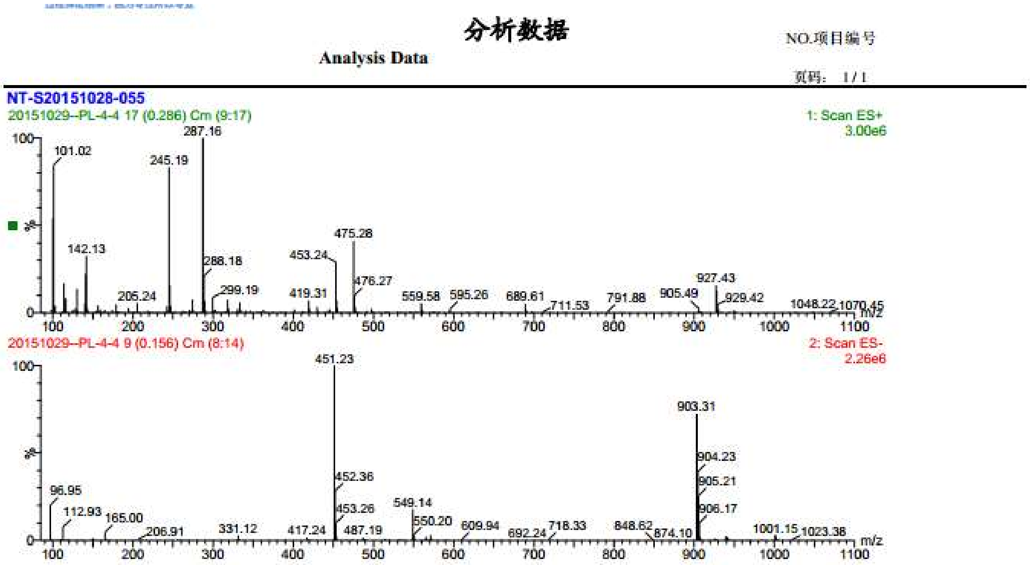

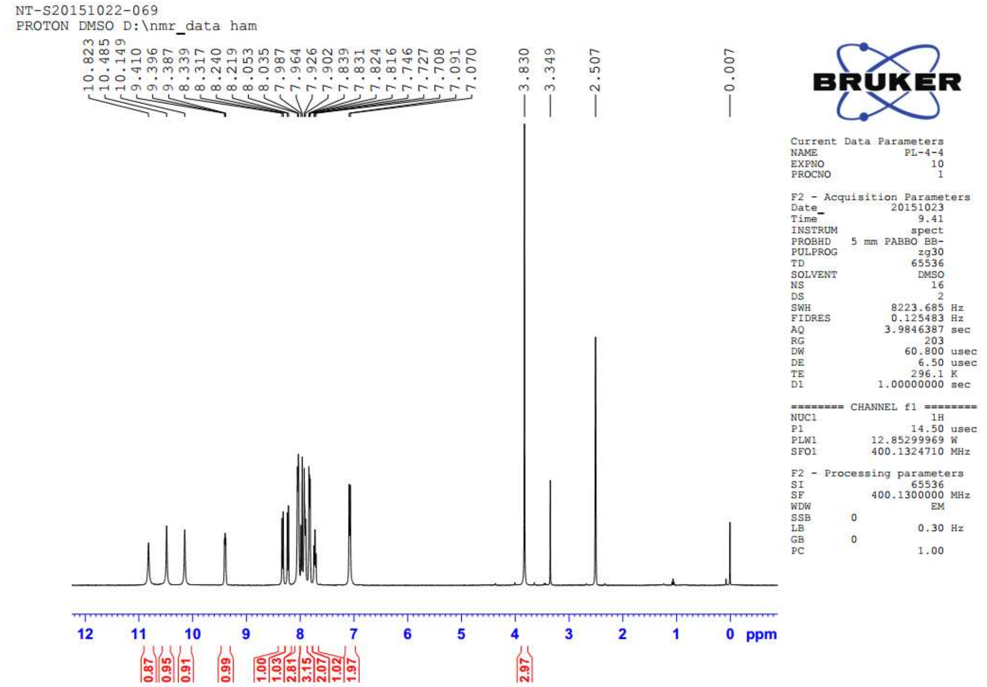

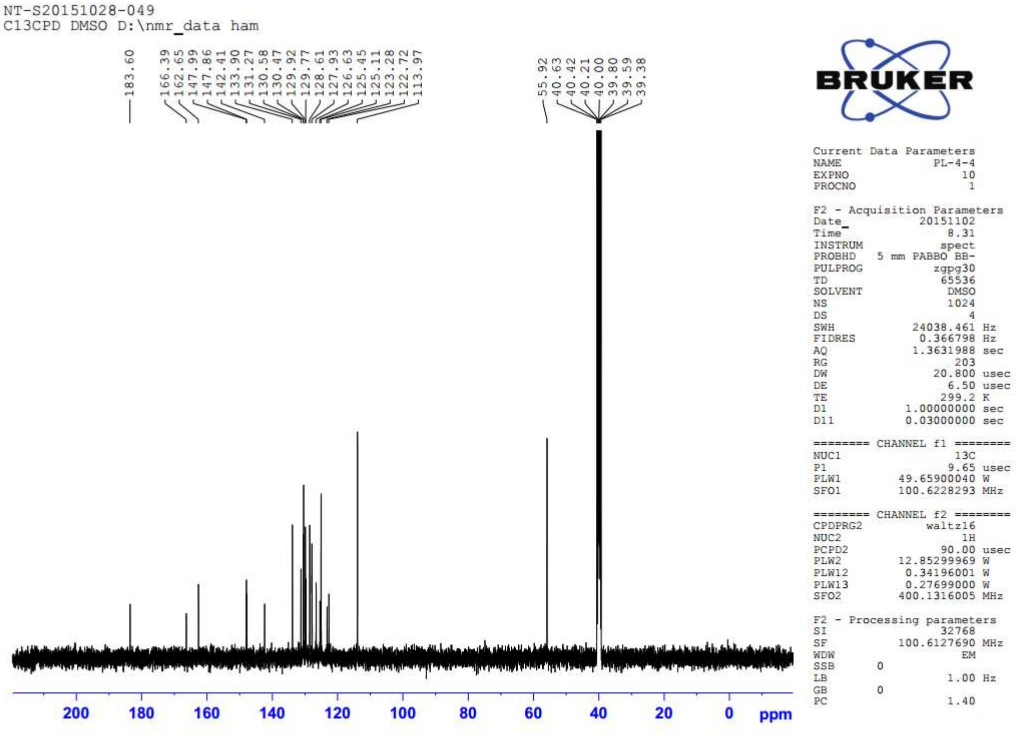

(5) The MS, ^{1}HNMR, ^{13}CNMR spectrum of **4e**

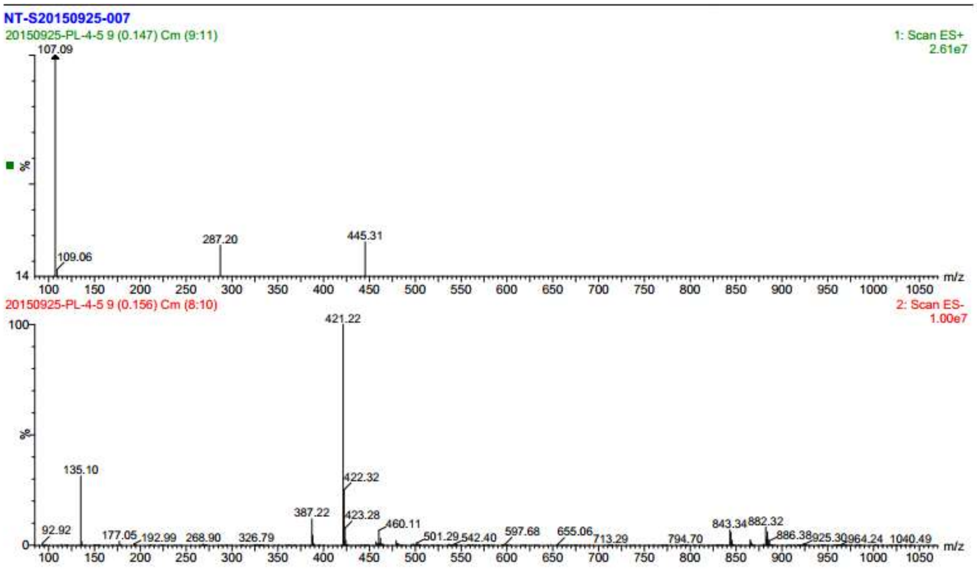
NT-S20150925-007
20150925-PL-4-5 9 (0.147) Cm (9:11)
1: Scan ES+
2.61e7
107.09
109.06
287.20
445.31
20150925-PL-4-5 9 (0.156) Cm (8:10)
2: Scan ES-
1.00e7
421.22
135.10
422.32
387.22
423.28
460.11
m/z

-1-

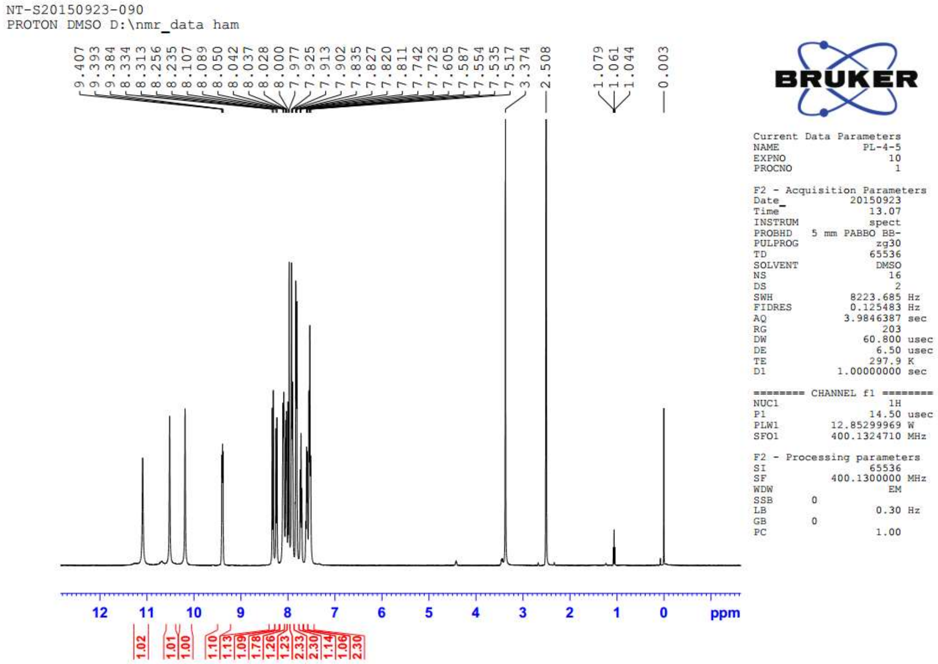
NT-S20150923-090
PROTON DMSO D:\nmr_data ham
BRUKER
Current Data Parameters
NAME PL-4-5
EXPNO 10
PROCNO 1
F2 - Acquisition Parameters
Date_ 20150923
Time 13.07
INSTRUM spect
PROBHD 5 mm PABBO BB-
PULPROG zg30
TD 65536
SOLVENT DMSO
NS 16
DS 2
SWH 8223.685 Hz
FIDRES 0.125483 Hz
AQ 3.9846387 sec
RG 203
DW 60.800 usec
DE 6.50 usec
TE 297.9 K
D1 1.00000000 sec
======== CHANNEL f1 ========
NUC1 1H
P1 14.50 usec
PLW1 12.85299969 W
SFO1 400.1324710 MHz
F2 - Processing parameters
SI 65536
SF 400.1300000 MHz
WDW EM
SSB 0
LB 0.30 Hz
GB 0
PC 1.00
ppm

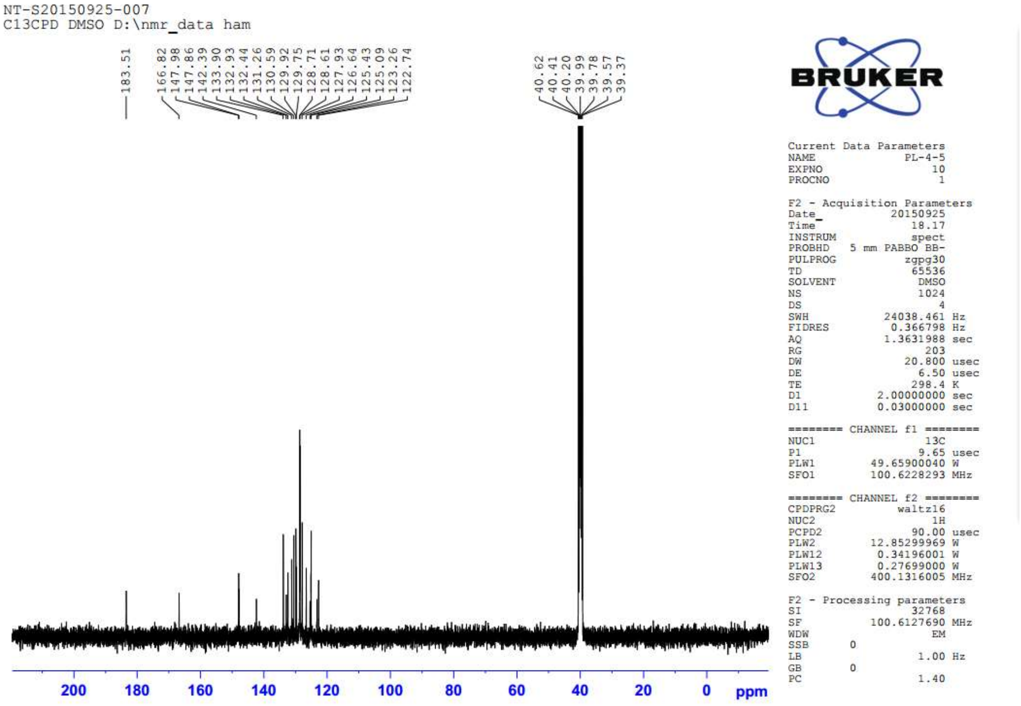
NT-S20150925-007
C13CPD DMSO D:\nmr_data ham
183.51
166.82
147.98
147.86
142.39
133.90
132.93
132.44
131.26
130.59
129.92
129.75
128.71
128.61
127.93
126.64
125.43
125.09
123.26
122.74
40.62
40.41
40.20
39.99
39.78
39.57
39.37
BRUKER
Current Data Parameters
NAME PL-4-5
EXPNO 10
PROCNO 1
F2 - Acquisition Parameters
Date_ 20150925
Time 18.17
INSTRUM spect
PROBHD 5 mm PABBO BB-
PULPROG zgpg30
TD 65536
SOLVENT DMSO
NS 1024
DS 4
SWH 24038.461 Hz
FIDRES 0.366798 Hz
AQ 1.3631988 sec
RG 203
DW 20.800 usec
DE 6.50 usec
TE 298.4 K
D1 2.00000000 sec
D11 0.03000000 sec
======== CHANNEL f1 ========
NUC1 13C
P1 9.65 usec
PLW1 49.65900040 W
SFO1 100.6228293 MHz
======== CHANNEL f2 ========
CPDPRG2 waltz16
NUC2 1H
PCPD2 90.00 usec
PLW2 12.85299969 W
PLW12 0.34196001 W
PLW13 0.27699000 W
SFO2 400.1316005 MHz
F2 - Processing parameters
SI 32768
SF 100.6127690 MHz
WDW EM
SSB 0
LB 1.00 Hz
GB 0
PC 1.40
200 180 160 140 120 100 80 60 40 20 0 ppm
